# Bioactive components and mechanisms of Chinese poplar propolis alleviates oxidized low-density lipoprotein-induced endothelial cells injury

**DOI:** 10.1186/s12906-018-2215-8

**Published:** 2018-05-03

**Authors:** Huasong Chang, Wenwen Yuan, Haizhu Wu, Xusheng Yin, Hongzhuan Xuan

**Affiliations:** 1 School of Life Science, Liaocheng University, Liaocheng, 252059 China; 2 Institute of Apiculture and bee product quality inspection of Shandong Province, Taian, 271000 China

**Keywords:** Propolis, Bioactive component, Oxidized low density lipoprotein, Human umbilical vein endothelial cells, Apoptosis

## Abstract

**Background:**

Propolis, a polyphenol-rich natural product, has been used as a functional food in anti-inflammation. However, its bioactive components and mechanisms have not been fully elucidated.

To discover the bioactive components and anti-inflammatory mechanism, we prepared and separated 8 subfractions from ethyl acetate extract of Chinese propolis (EACP) and investigated the mechanism in oxidized low density lipoprotein (ox-LDL) induced human umbilical vein endothelial cells (HUVECs) damage.

**Methods:**

Eight subfractions were prepared and separated from ethyl acetate extract of Chinese propolis (EACP) with different concentrations of methanol-water solution, and analysed its chemical constituents by HPLC-DAD/Q-TOF-MS. Then 80% confluent HUVECs were stimulated with 40 μg/mL ox-LDL. Cell viability and apoptosis were evaluated by Sulforhodamine B (SRB) assay and Hoechst 33,258 staining, respectively. Levels of caspase 3, PARP, LC3B, p62, p-mTOR, p-p70S6K, p-PI3K, p-Akt, LOX-1 and p-p38 MAPK were assessed by western blotting and immunofluorescence assay, respectively. Reactive oxygen species (ROS) and mitochondrial membrane potential (MMP) were measured with fluorescent probes.

**Results:**

Each subfraction exhibited similar protective effect although the contents of chemical constituents were different. EACP attenuated ox-LDL induced HUVECs apoptosis, depressed the ratio of LC3-II/LC3-I and enhanced the p62 level. In addition, treatment with EACP also activated the phosphorylation of PI3K/Akt/mTOR, and deactivated the level of LOX-1 and phosphorylation of p38 MAPK. The overproduction of ROS and the damage of MMP were also ameliorated after ECAP treatment.

**Conclusions:**

These findings indicated that the bioactive component of propolis on anti-inflammatory activity was not determined by a single constituent, but a complex interaction including flavonoids, esters and phenolic acids. EACP attenuated ox-LDL induced HUVECs injury by inhibiting LOX-1 level and depressed ROS production against oxidative stress in ox-LDL induced HUVECs, further to activate PI3K/Akt/mTOR pathway and deactivate p38 MAPK to inhibit apoptosis and autophagy, which provide novel insights into the potential application of propolis on modulating chronic inflammation.

## Background

Atherosclerosis, a complex chronic inflammatory and metabolic disease, has become the major cause for morbidity and mortality worldwide [1]. Oxidized low density lipoprotein (ox-LDL) is now considered to play a critical role in the pathogenesis of atherosclerosis by inducing intracellular lipid accumulation and foam cell formation [2]. During this process, ox-LDL up-regulates the expression of adhesion molecules and recruits the monocytes to the sub endothelial space, leading to the impairment of endothelial cells and decrease of antioxidant capability [3]. Excessive reactive oxygen species (ROS) production stimulates the detrimental modification of vital intracellular macromolecules, such as lipids, proteins, and DNA, resulting in macrophage apoptosis [4]. Protecting the endothelium against ox-LDL-induced endothelial apoptosis and the modulation of intracellular ROS levels has been considered a novel a therapeutic strategy for atherosclerosis.

Propolis is a resinous material that honey bee *(Apis mellifera* L.) collect from various plant-derived substances [5, 6]. It has widely used as a functional food since ancient time for its widely pharmacological activities, such as antimicrobial, antioxidant, anti-inflammatory, immunomodulatory, and cardioprotective effects [7]. It is a well-established fact that propolis has good anti-inflammatory effect [8]. Recent study reported that polyphenol-rich propolis extracts strengthened intestinal barrier by activating AMPK and ERK signaling in Caco-2 cells [9]. Another report indicated that propolis exhibited strong free-radical scavenging activity and significant in vitro anti-inflammatory effects by modulating key inflammatory mediators of mRNA transcription, inhibiting the production of specific inflammatory cytokines, and blocking the activation of nuclear factor NF-κB [10].

We and other researchers have reported the protective effects of propolis on regulation of dyslipidemia, which is known resulting in the genesis and progression of atherosclerosis [11–13]. However, the bioactive components of Chinese propolis on the protective activity of endothelial cellular injury are still unclear, since the chemical constituents of propolis are very complex. There are more than 300 constituents in propolis, mainly flavonoids and phenolic acids [5, 14]. More importantly, the mechanisms of propolis on modulating chronic inflammatory diseases are still not fully elucidated.

This study aimed to determine the bioactive constituents and discover possible mechanisms of Chinese poplar propolis in ox-LDL-induced human umbilical vein vascular endothelial cells (HUVECs) injury and discussed its potential application in chronic inflammatory diseases.

## Methods

### Materials

Dulbecco’s modified Eagle’s medium (DMEM) was obtained from Gibco (USA). Fetal bovine serum (FBS) was from Hyclone Lab Inc. (USA). Sulforhodamine B (SRB), Hoechst 33,258, 2′,7′-dichlorodihydrofluorescin (DCHF) and JC-1 were from Sigma Co. (USA). Primary antibodies against β-actin, PI3K and p-PI3K were from Santa Cruz Biotechnology (USA). Primary antibody against caspase 3, PARP, LC3B, p70S6K, p-p70S6K, p-mTOR, mTOR, Akt, p-Akt, p38 MAPK, p-p38 MAPK and secondary antibody (horseradish peroxidase) were from Cell Signaling Technology (USA). Primary antibody against p62 was from BD Transduction Laboratories. Primary antibody against LOX-1 was from ABclonal (USA). Wortmannin was obtained from Selleck (USA). Ox-LDL was purchased from Beijing Xiesheng Biotechnology (China). All other reagents were ultrapure grade.

### Preparation of ethyl acetate extract of Chinese propolis (EACP)

Chinese propolis was obtained from colonies of honeybees, *A. mellifera* L., in Shandong province of north China in 2010 and the main plant origin was poplar (*Populus* sp.). Chinese propolis 0.25 kg was frozen, milled and extracted with boiling water. The water extract was filtered, and the remaining part was extracted by ethyl acetate, then the bioactive components of ethyl acetate fraction of Chinese propolis (EACP) were separated and enriched on a glass column (30 cm × 5 cm I.D., 1BV = 500 mL) wet-packed with 200 g of octadecyl silane bonded silica (40–60 μm, purchased from YMC CO., LTD). 20 g of the cream was dissolved in water and then loaded on the column and eluted with 6 BV of 40%, 50%, 60%, 65%, 70%, 75%, 80%, and 90% methanol-water solution, successively. The eluent from the tail end of the column was collected at 200 mL intervals and analyzed by high-pressure liquid chromatography (HPLC). The eluent with the same composition was collected according to HPLC analysis. Finally, the fractions eluted with 40%, 50%, 60%, 65%, 70%, 75%, 80%, and 90% methanol-water solution gave subfractionI, II, III, IV, V, VI, VII and VII, respectively. The chemical constituents of eight subfractions were analyzed by HPLC-DAD/Q-TOF-MS analysis as previously described [15, 16].

### Cell culture

HUVECs were gifted by Atherosclerosis Research Institute of Taishan Medical University of China purchased from ATCC. Cells were cultured in DMEM medium supplemented with 10% (*v*/v) FBS at 37 °C in a humidified incubator with 5% CO_2_.

### Cell viability assay

SRB assay was used to determine cell viability. Briefly, cells were precipitated with 100 μL 10% trichloroacetic acid for 1 h at 4 °C. Then cells were added 50 μL of 0.4% (*W*/*V*) SRB solution to each well for 20 min at room temperature. After that, cells were washed the plates five times with 1% acetic acid and subsequently added 100 μL of 10 mM Tris base to dissolve the bound dye. Mixed 5 min on a microtiter plate shaker and read optical densities at the wavelength of 492 nm using microplate spectrophotometer.

The viability (%) was expressed as (OD of treated group/OD of ox-LDL group) × 100%. The viability of the ox-LDL group was set at 100%.

### Acridine orange staining

Acridine orange staining was used to test the morphological changes of nuclei. At 6 h, cells were stained with 5 μg/mL acridine orange at room temperature for 5 min and observed under a laser scanning confocal microscopy (Olympus FV1200, Japan).

### Hoechst 33,258 staining

Hoechst 33,258 staining was used to observe apoptotic morphology. At 6 h, cells in all groups were stained with 10 μg/mL Hoechst 33,258 for 15 min. Cells were gently washed with phosphate buffered saline (PBS) once. Nuclear condensation and fragmentation were observed under a TE2000S fluorescence microscope (Nikon, Japan).

### Measurement of reactive oxygen species (ROS) production

Intracellular ROS levels were measured with 2′,7′-dichlorodihydrofluorescein (DCHF), which could be rapidly oxidized into a highly fluorescent dichlorofluorescin (DCF) by intracellular ROS on entry into cells. Cells were treated for 6 h, then washed with basal DMEM medium for 5 min and incubated with DCHF 0.5 ml at 37 °C for 30 min. After washing the cells three times with basal DMEM medium, the fluorescence was monitored with a confocal laser scanning microscope (Olympus FV1200, Japan). The photographs were representatives of three independent experiments. Results were shown as the relative fluorescence intensity ratio compared with ox-LDL group.

### Measurement of mitochondrial membrane potential

Mitochondrial membrane potential (MMP) assay was performed by JC-1 aggregates that are formed as a function of inner mitochondrial membrane potential [17]. After treatment, the cells were incubated in a humidified incubator at 37 °C with 10 μg/mL JC-1 for 15 min. Then cells were washed with basal DMEM medium three times, the fluorescence was monitored with a confocal laser scanning microscope (Olympus FV1200, Japan). Results were shown by ratio of red to green fluorescence as compared with the ox-LDL group.

### Immunofluorescence assay

Immunofluorescence assay was performed as previously method [18]. Cells were fixed in 4% paraformaldehyde (*w*/*v*) for 15 min at room temperature and blocked in 5% donkey serum (*v*/v). After adding the primary (1:100) and second antibodies (1:200) (FITC-IgG), a laser scanning confocal microscope (Olympus FV1200, Japan) was used for fluorescence detection. Analysis was used the Image-Pro Plus software (USA). Images are representative of three independent experiments.

### Western blotting analysis

Western blotting assay was performed by a previous method [18]. Cells were washed three times with cold PBS and lysed in lysis buffer with protease inhibitors at ice. Thirty micrograms of protein were separated by 12% SDS-PAGE and electro-blotted to a polyvinylidene fluoride (PVDF) membrane using a semi-dry blotting apparatus (Bio-Rad, USA). After adding the primary (1:1000) and second antibodies (1:3000–5000), the bands were visualized using an enhanced chemiluminescent detection kit (Thermo Electron Corp., USA). β-actin was used as loading control.

### Statistical analysis

Data are from at least three independent experiments and expressed as means ± S.E.M. Statistical analysis involved the paired Student *t* test and ANOVA with SPSS Ins (PASW Statistics 18). Differences were considered statistically significant at *P* < 0.05.

## Results

### Effect of 8 subfractions and EACP on cell viability in ox-LDL induced HUVECs injury

**Fig. 1:**
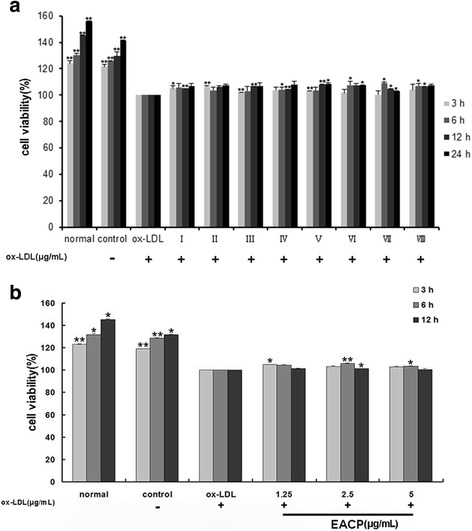
Effects of 8 subfractions separated from ethyl acetate extract of Chinese propolis (EACP) and EACP on cell viability in ox-LDL induced HUVECs injury. **a**, Effects of the 8 subfractions separated from EACP (2.5 μg/mL) on cell viability in ox-LDL (40 μg/mL) induced HUVECs for 3, 6, 12 and 24 h. **b**, Effects of the EACP (1.25, 2.5 and 5 μg/mL) on cell viability in ox-LDL induced HUVECs at 3, 6 and 12 h. Cell viability was tested by SRB assay and illustrated in column figures. (^*^P < 0.05, ^**^P < 0.01 vs ox-LDL group, *n* = 3). Data are means ± S.E.M.

To discover the bioactive components of EACP in HUVECs stimulated by ox-LDL, we first tested the cell viability of the 8 subfractions separated from EACP on ox-LDL induced HUVECs. Cell proliferation was obviously inhibited after ox-LDL (40 μg/mL) treatment, and an interesting fact was that all 8 subfractions exhibited similar protection effects on ox-LDL induced HUVECs (Fig. 1a), although the chemical constituents were different (Table 1). We deduced that the bioactive components of propolis on the protection effects in ox-LDL induced HUVECs were not determined by a single component. Thus we used EACP for the following experiments, and then investigated the effects of EACP (1.25, 2.5, 5 μg/mL) on cell proliferation in ox-LDL-induced HUVECs at 3, 6 and 12 h. The results indicated EACP significantly enhanced cell viability at the concentration of 2.5 μg/mL for 6 h (Fig. 1b), but the increased cell proliferation was lower than challenged with each subfraction.

**Table 1:** HPLC-DAD/Q-TOF-MS analysis on eight subfractions of EACP

Compounds	Content (μg/mL)								
	I	II	III	IV	V	VI	VII	VII	EACP
caffeic acid	–	–	–	–	–	–	0.22	0.25	1.11
*p*-Coumaric acid	–	–	–	–	–	–	1.45	0.22	1.33
ferulic Acid	–	–	–	–	–	–	0.03	0.13	0.95
isoferulic acid	66.28	3.78	7.35	22.42	38.47	5.98	0.01	0.07	0.86
3,4-dihydroxybenzoic acid	19.03	8.11	4.38	2.34	6.71	9.05	0.09	0.12	–
trans-Cinnamic acid	–	–	–	–	–	–	0.03	0.33	–
phenethyl caffeate	–	1.04	6.72	9.02	4.04	28.67	9.51	13.12	30.00
apigenin	–	128.22	26	14.56	3.53	45.2	47.01	86.82	94.09
chrysin	–	12.8	8.57	0.22	0.39	78.39	151.52	276.93	95.54
quercetin	–	48.29	66.54	10.25	–	–	–		7.18
kaempferol	–	25.44	90.48	49.35	12.17	12.72	2.39	6.15	18.39
galangin	40.56	1.59	92.11	24.09	20.83	33.54	5.32	9.53	28.89
pinocembrin	–	1.35	1.99	1.18	17.09	–	33.50	47.10	84.29
pinobanksin	47.46	79.29	193.88	263.47	114	70.74	11.18	15.59	44.17
luteolin	–	63.46	93.06	20.82	12.03	–	0.42	–	–

### EACP inhibited apoptosis and autophagy in ox-LDL induced HUVECs

**Fig. 2:**
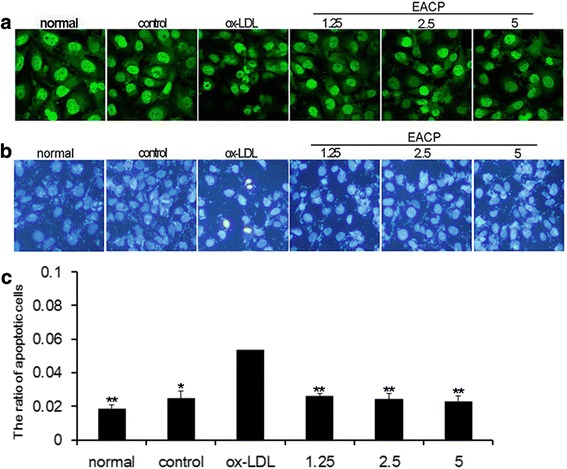
EACP inhibited ox-LDL induced apoptosis in ox-LDL induced HUVECs injury. **a**, AO staining showed treatment with EACP (1.25, 2.5 and 5 μg/mL) depressed nuclear condensation and fragmentation in ox-LDL induced HUVECs. **b**, Hoechst 33,258 staining suggested that treatment with EACP inhibited apoptosis in ox-LDL induced HUVECs. **c**, Quantification of relative apoptosis rate in ox-LDL induced HUVECs. (^*^P < 0.05, ^**^P < 0.01 vs ox-LDL group, *n* = 3). Data are means ± S.E.M.

**Fig. 3:**
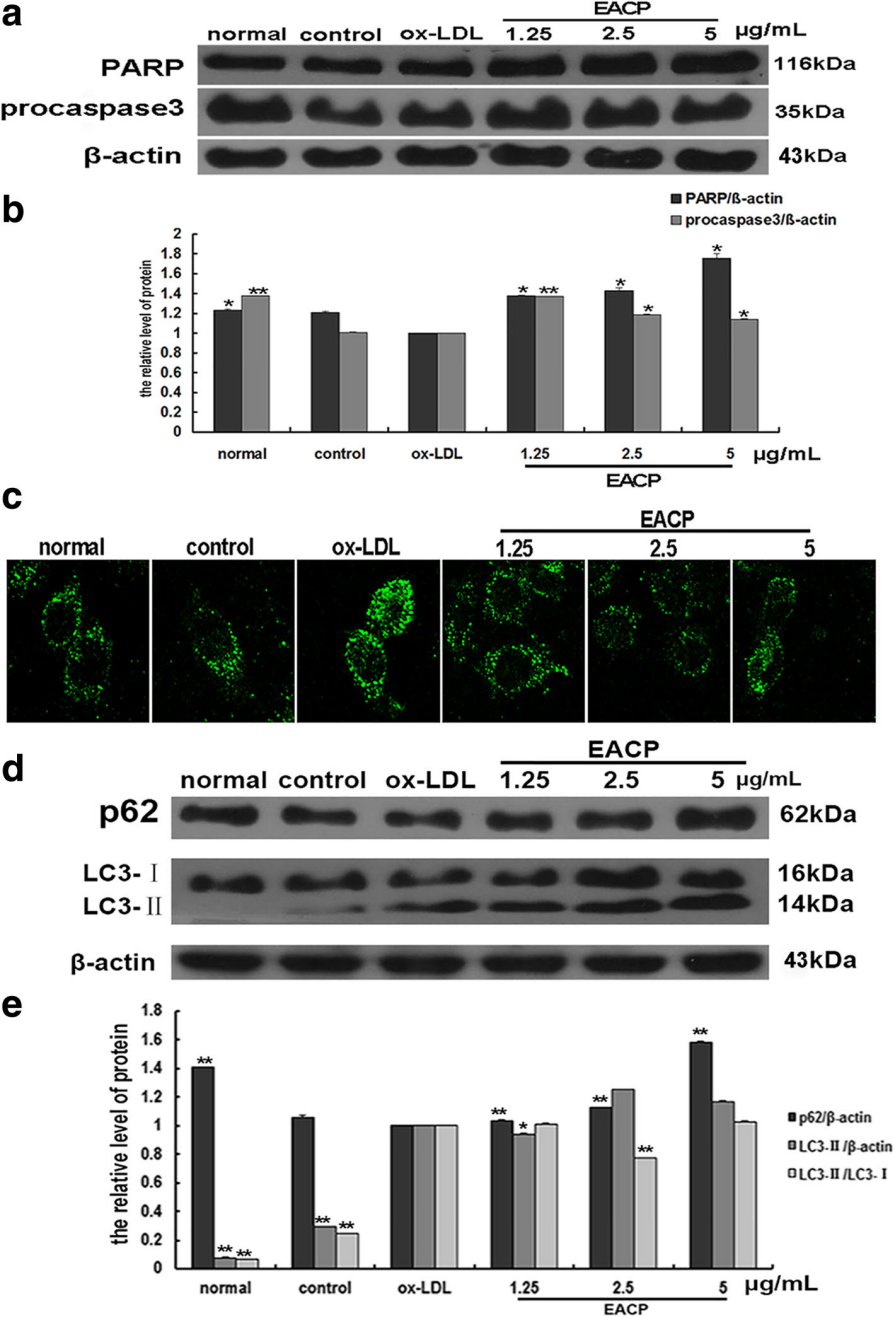
EACP inhibited the levels of caspase 3, PARP cleavage, the ratio of LC3-II/LC3-I and enhanced the p62 level in ox-LDL induced HUVECs at 6 h. **a**, Expression of procaspase 3 and full length PARP after treatment with EACP in ox-LDL induced HUVECs; **b**, Quantification of relative expression quantity in ox-LDL induced HUVECs at 6 h; **c**, Cells were stained with anti-LC3B antibody for immunostaining. Immunofluorescence graphs showed a decrease of endogenous punctuate LC3 after treatment with EACP; **d**, Expression of p62 and LC3B in ox-LDL induced HUVECs; **e**, Quantification of relative expression quantity in ox-LDL induced HUVECs at 6 h, respectively. (^*^P < 0.05, ^**^P < 0.01 vs ox-LDL group, *n* = 3). Data are means ± S.E.M.

We then evaluated the effects of EACP (1.25, 2.5 and 5 μg/mL) on apoptosis. AO and Hoechst 33,258 staining showed that challenged with EACP obviously inhibited ox-LDL induced nuclear condensation, fragmentation and apoptosis in HUVECs (Fig. 2). Western blotting results also indicated that treatment with EACP evidently attenuated apoptosis by inhibiting caspase 3 level and PARP cleavage (Fig. 3a, b).

In order to study other possible protection mechanism of EACP on ox-LDL stimulated HUVECs, we further investigated the effects of EACP on autophagy. The results from immunofluorescence test showed a decrease of endogenous punctuate LC3 after treatment with EACP (Fig. 3c), and western blotting results also indicated that EACP significantly decreased the ratio of LC3-II/LC3-I, and the level of p62 evidently enhanced after EACP treatment in ox-LDL stimulated HUVECs (Fig. 3d).

### EACP inhibited ox-LDL-decreased mTOR activity in HUVECs

**Fig. 4:**
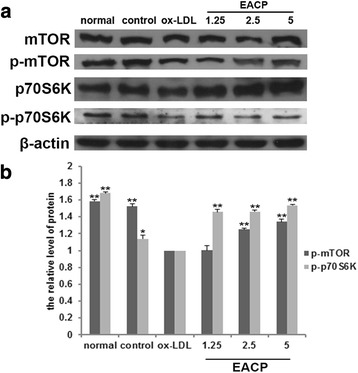
EACP inhibited ox-LDL-decreased mTOR activity in HUVECs. **a**, Expression of mTOR, p-mTOR, p70S6K, p-p70S6K after treatment with EACP in ox-LDL induced HUVECs at 6 h; **b**, Quantification of relative expression quantity of p-mTOR and p-p70S6K in ox-LDL induced HUVECs at 6 h. (^*^P < 0.05, ^**^P < 0.01 vs ox-LDL group, *n* = 3). Data are means ± S.E.M.

In order to clarity how EACP affected autophagy in ox-LDL induced HUVECs. We observed that ox-LDL inhibited the phosphorylation of mTOR and its downstream targets p70S6K, and EACP treatment reversed the ox-LDL-inhibited phosphorylation of mTOR and p70S6K, suggesting that EACP activating mTOR. Furthermore, EACP inhibiting autophagy was an mTOR-dependent manner (Fig. 4).

### EACP promoted phosphorylated levels of PI3K and Akt in ox-LDL induced HUVECs

**Fig. 5:**
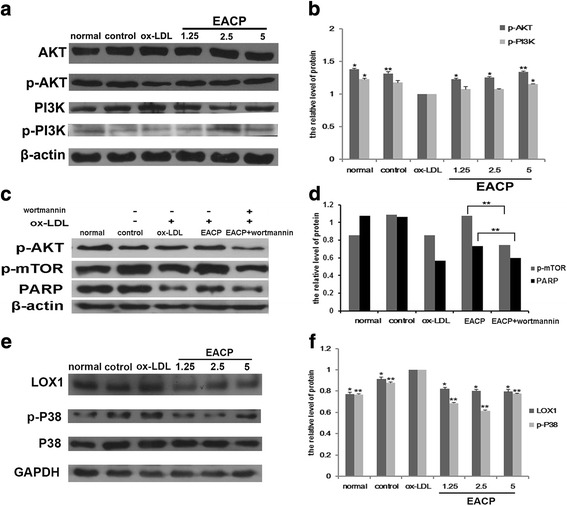
EACP activated PI3K/Akt and depressed LOX-1/ p38 MAPK in ox-LDL induced HUVECs at 6 h. **a**, Expression of Akt, p-Akt, PI3K, p-PI3K after treatment with EACP in ox-LDL induced HUVECs at 6 h, respectively; **b**, Quantification of relative expression quantity of p-Akt and p-PI3K in ox-LDL induced HUVECs at 6 h, respectively. **c**, Expression of p-Akt, p-mTOR, full length PARP after treatment with EACP combined with wortmannin. **d**, Quantification of relative expression quantity of p-mTOR and full length PARP after treatment with EACP combined with wortmannin. **e**, Expression of LOX-1, p38 MAPK, p-p38 MAPK after treatment with EACP in ox-LDL induced HUVECs at 6 h, respectively; **f**, Quantification of relative expression quantity of LOX-1 and p-p38 MAPK in ox-LDL induced HUVECs at 6 h, respectively. (^*^P < 0.05, ^**^P < 0.01 vs ox-LDL group, *n* = 3). Data are means ± S.E.M.

Previous study indicated that activation of mTOR inhibited both apoptosis and autophagy under oxidative stress via activating Akt [19]. It is well known that activation of PI3K/Akt is vital for cells to suppress apoptosis and promote cell survival [20]. To investigate whether PI3K/Akt signaling was involved in the protective effects of propolis in ox-LDL induced HUVECs by activating its down stream molecule mTOR, we tested the levels of p-PI3K and p-Akt by western blotting. As shown in Fig. 5a, phosphorylated levels of Akt and PI3K in cells treated with EACP obviously increased compared with the ox-LDL group. Furthermore, the level of p-mTOR was depressed after inhibiting the activity of p-Akt by its inhibitor wortmannin, and the inhibition effect of EACP on apoptosis was also reversed when the activity of p-Akt was inhibited (Fig. 5c).

### EACP decreased the LOX1 level and phosphorylation of p38 MAPK in ox-LDL induced HUVECs

Many reports have demonstrated that LOX-1 plays an important role in the mediation of the effects of ox-LDL on endothelial biology [21]. Activation of LOX-1 by ox-LDL initiates the intracellular signaling pathways, leading to the sequential phosphorylation of a series of protein kinases, tyrosine kinases, and mitogen-activated protein kinases (MAPK). We found that challenged with ox-LDL, the levels of LOX-1 and phosphorylation of p38 MAPK obviously enhanced. Treatment with EACP significantly attenuated LOX-1 level and phosphorylation of p38 MAPK in ox-LDL induced HUVECs (Fig. 5e).

### EACP decreased the production of ROS and protected MMP in ox-LDL induced HUVECs

**Fig. 6:**
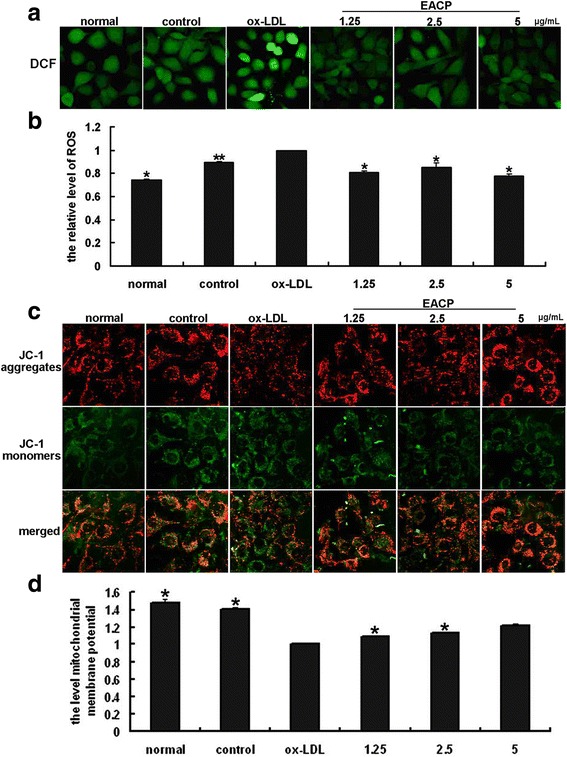
EACP decreased the production of ROS and protect mitochondrial membrane potential (MMP) in ox-LDL induced HUVECs. **a**, Fluorescent micrographs of ROS obtained in ox-LDL induced HUVECs at 6 h. **b**, Quantification of relative quantity of ROS in ox-LDL induced HUVECs at 6 h. Values represent the relative fluorescent intensity per cell determined by laser scanning confocal microscopy. **c**, Fluorescent micrographs of mitochondrial membrane potential obtained in ox-LDL induced HUVECs at 6 h. **d**, Quantification of relative fluorescent intensity per cell determined by laser scanning confocal microscopy. Values represent as ratio of red to green fluorescence. (^*^P < 0.05, ^**^P < 0.01 vs ox-LDL group, *n* = 3). Data are means ± S.E.M.

Activation of LOX-1 by ox-LDL initiates the intracellular signaling pathways. LOX-1 promotes the generation of ROS. As shown in Fig. 6, treatment with ox-LDL led to an obvious increase accumulation of intracellular ROS and damage of MMP. After treatment with EACP, the production of ROS in ox-LDL stimulated HUVECs evidently alleviated and the MMP damage was also ameliorated after EACP treatment, suggesting protection effects of EACP against ox-LDL-induced damage by decreasing ROS and elevated MMP (Fig. 6).

## Discussion

In this study, we detailed studied the bioactive components and mechanism of propolis on ox-LDL induced HUVECs injury. Importantly, we firstly discovered the bioactive components of propolis on the protection effects in ox-LDL induced HUVECs injury were not determined by a single compound, and provided new insights to elucidate the mechanisms of propolis on chronic inflammation including atherosclerosis by activating PI3K/Akt/mTOR signaling pathway, inhibiting LOX-1/p38 MAPK level, depressing ROS production and protecting MMP to inhibit apoptosis and autophagy.

Propolis is an active mixture in modulating inflammation [11]. However, its bioactive components are still unclear. We separated 8 subfractions from EACP, and tested the effects of each subfraction on ox-LDL induced HUVECs injury and analysed the 15 constituents of each subfraction. Our finding indicated that although the contents of 15 constituents of each subfraction were different, they exerted similar protect effects on ox-LDL induced HUVECs injury. Based on these, we deduced that it was not a single constituent to exert the protection effects, and there might be a synergy and an antagonism effect among these constituents of propolis, which as a whole contributed to the protection effects in ox-LDL induced HUVECs injury. Compared with the bioactive components of antitumor activity of Chinese poplar propolis, the anti-inflammatory bioactive components are different with that of antitumor, which depends on flavonoids and esters, and phenolic acids have no effects on antitumor [22]. However, phenolic acids might exert their functions in anti-inflammatory activity.

Besides, we also found treatment with EACP effectively attenuated ox-LDL induced HUVECs apoptosis by increasing cell viability, alleviating apoptosis by inhibiting the level of caspase 3 and PARP cleavage.

Akt, a downstream effector of PI3K, encourages cell survival in response to various death incentives [23]. The activation of Akt involves its phosphorylation on threonine 308 and on serine 473 by PI3K [24]. Evidence suggests that the PI3K/Akt pathway shows an important role in inhibiting ROS-induced endothelial damage by scavenging superoxide anion [25, 21]. Propolis is a kind of polyphenol-rich material, and exerts excellent antioxidant activity [26, 27]. Our study showed that ox-LDL induced ROS production and MMP damage, and treatment with propolis evidently decreased ROS and elevated MMP, suggesting a good antioxidant effect on ox-LDL induced endothelial injury. Furthermore, the phosphorylation of PI3K/Akt obviously enhanced after EACP treatment in ox-LDL induced HUVECs, suggesting the relationship between the decreasing the ROS level and activating the levels of phosphorylation of PI3K/Akt of propolis treatment in endothelial impairment.

mTOR is the downstream molecule of PI3K/Akt which participates in controlling cell proliferation, protein synthesis, autophagy, and metabolism [28]. There were different reports on the roles of mTOR on atherosclerosis. Some reports indicated that mTOR inhibitors induced autophagy in macrophages, which might have potential ability of plaque-stabilizing [29–31]. Thus mTOR inhibitors are currently being used to treat atherosclerosis in clinical trials [32]. However, other studies showed that drug-induced macrophage autophagy might lead to a pro-inflammatory response and post-autophagic necrosis [33]. And they suggested that the activation of mTOR could inhibit both apoptosis and autophagy under oxidative stress via activating Akt in differentiated ECs [19]. Peng et al. (2014) also indicated that activating mTOR might be a promising therapeutic strategy to prevent or treat atherosclerosis and other cardiovascular diseases by protecting the endothelium [34]. Our results showed that Chinese poplar propolis activated mTOR and its downstream p70S6K to inhibit apoptosis and autophagy. We also demonstrated that activation of PI3K/Akt leading to the activation of p- mTOR after Chinese poplar propolis treatment in ox-LDL stimulated HUVECs could be depressed by Akt inhibitor -wortmannin, and protection effect of Chinese poplar propolis was directly related with PI3K/Akt/mTOR signaling pathway, which might provide a new insight for understanding the protective effects of propolis against endothelium apoptosis.

LOX-1, the receptor for ox-LDL, was highly expressed in atherosclerosis, diabetes, hypertension and other diseases [35]. Activation of LOX-1 by ox-LDL initiates the intracellular signaling pathways. Increasing evidence shows that LOX-1 promotes the generation of ROS. The ROS over-production leads to the sequential phosphorylation of a series of protein kinases, tyrosine kinases, and mitogen-activated protein kinases (MAPK) [3]. A recent report showed that LOX-1 signaling and its down-stream p38 MAPK pathways were participated in the ox-LDL induced endothelial cells injury. It has reported that various intracellular signal pathways are involved in the ox-LDL induced endothelial cells apoptosis, including phosphorylation and activation of ERK1/2, JNK, and p38 MAPKs. The blocking of p38 MAPK activation protects several cells against apoptosis. Our study showed that Chinese poplar propolis alleviated ox-LDL stimulated HUVECs injury via inhibiting the expression of LOX-1 and phosphorylation of p38 MAPK, suggesting that LOX-1/p38 MAPK might involve in the protection effects of propolis.

## Conclusion

Taken together, the bioactive components of Chinese propolis on protecting ox-LDL-induced endothelial injury were not determined by a single component, and there might be a “bioactive components”. Chinese poplar propolis alleviated ox-LDL induced HUVECs injury by activating PI3K/Akt/mTOR signaling pathway, inhibiting LOX-1/p38 MAPK level, depressing ROS production and protecting MMP to inhibit apoptosis and autophagy. Our study provides novel insights into the potential applications of propolis for the treatment of chronic inflammation including atherosclerosis.

## Abbreviations

DCHF: 2′,7′-dichlorodihydrofluorescin
DMEM: Dulbecco’s modified Eagle’s medium
EACP: ethyl acetate extract of Chinese propolis
FBS: Fetal bovine serum
HPLC: high-pressure liquid chromatography
HUVECs: human umbilical vein endothelial cells
MMP: mitochondrial membrane potential
ox-LDL: oxidized low density lipoprotein
PBS: phosphate buffered saline
PVDF: polyvinylidene fluoride
ROS: Reactive oxygen species
SRB: Sulforhodamine B
